# Socio-economic variation in CT scanning in Northern England, 1990-2002

**DOI:** 10.1186/1472-6963-12-24

**Published:** 2012-01-27

**Authors:** Mark S Pearce, Jane A Salotti, Kieran McHugh, Kwang Pyo Kim, Alan W Craft, Jay Lubin, Elaine Ron, Louise Parker

**Affiliations:** 1 Institute of Health and Society, Newcastle University, Sir James Spence Institute, Royal Victoria Infirmary, Newcastle upon Tyne, NE1 4LP, UK; 2 Great Ormond Street Hospital for Children NHS Trust, London, WC1N 3JH, UK; 3 Department of Nuclear Engineering, Kyung Hee University, Gyeonggi-Do, Republic of Korea; 4 Northern Institute of Cancer Research, Newcastle University, Sir James Spence Institute, Royal Victoria Infirmary, Newcastle upon Tyne, NE1 4LP, UK; 5 Division of Cancer Epidemiology and Genetics, National Cancer Institute, 6120 Executive Boulevard, Bethesda, Maryland, 20892, USA; 6 Deceased; 7 Department of Medicine and Paediatrics, Population Cancer Research Program, Dalhousie University, Halifax, Nova Scotia, Canada; 8 Cancer Care Nova Scotia, Halifax, Nova Scotia, Canada

## Abstract

**Background:**

Socio-economic status is known to influence health throughout life. In childhood, studies have shown increased injury rates in more deprived settings. Socio-economic status may therefore be related to rates of certain medical procedures, such as computed tomography (CT) scans. This study aimed to assess socio-economic variation among young people having CT scans in Northern England between 1990 and 2002 inclusive.

**Methods:**

Electronic data were obtained from Radiology Information Systems of all nine National Health Service hospital Trusts in the region. CT scan data, including sex, date of scan, age at scan, number and type of scans were assessed in relation to quintiles of Townsend deprivation scores, obtained from linkage of postcodes with census data, using χ^2 ^tests and Spearman rank correlations.

**Results:**

During the study period, 39,676 scans were recorded on 21,089 patients, with 38,007 scans and 19,485 patients (11344 male and 8132 female) linkable to Townsend scores. The overall distributions of both scans and patients by quintile of Townsend deprivation scores were significantly different to the distributions of Townsend scores from the census wards included in the study (p < 0.0001). There was a significant association between type of scan and deprivation quintile (p < 0.0001), primarily due to the higher proportions of head scans in the three most deprived quintiles, and slightly higher proportions of chest scans and abdomen and pelvis scans in the least deprived groups. There was also a significant association (p < 0.0001) between the patient's age at the time of the CT scan and Townsend deprivation quintiles, with slightly increasing proportions of younger children with increasing deprivation. A similar association with age (p < 0.0001) was seen when restricting the data to include only the first scan of each patient. The number of scans per patient was also associated with Townsend deprivation quintiles (p = 0.014).

**Conclusions:**

Social inequalities exist in the numbers of young people undergoing CT scans with those from deprived areas more likely to do so. This may reflect the rates of injuries in these individuals and implies that certain groups within the population may receive higher radiation doses than others due to medical procedures.

## Background

Socio-economic variation in health is well known across all age groups [1]. Children from disadvantaged groups have been repeatedly shown to be more likely to suffer injuries requiring presentation at a hospital in the UK [2-6]. This raises the prospect of socio-economic status being related to frequency of undergoing certain medical procedures, such as computed tomography (CT) scans.

CT scans represent an indispensable, sometimes life-saving diagnostic tool, for which new clinical applications continue to be identified. Since the introduction of CT scanning in medicine in the early 1970s, its use has increased rapidly. In the UK, the overall frequency of CT scanning increased by 39% between 1997/1998 and 2001/2002, whereas the frequency of conventional radiographic examinations increased by only 1% during the same period [7]. By 2008, the number of CT examinations in England (population 51 million) had risen to over 3 million per year [8]. We recently reported a doubling, between 1993 and 2002 inclusive, in the use of CT scans, in patients aged under 22 years, in Northern England [9].

Whilst the benefit to patients of having a CT scan can be substantial, the relatively high radiation doses associated with CT have given rise to growing concerns, in particular, a possible increase in future cancer risk [10-12]. Radiation dose from CT imaging is much higher than that from conventional x-ray, with effective doses per routine chest CT scans hundreds of times higher than that per chest radiograph [13]. Children can receive much higher radiation doses than necessary if scanned using adult CT settings [14]. Younger patients are more susceptible to the effects of radiation, partly due to their longer post-irradiation life expectancy and to potentially greater tissue damage, relative to adults, for equivalent radiation exposures [10]. Trauma is a common clinical indication for a young patient to undergo a CT scan. Given the relatively high radiation doses involved in CT scans, this study, as part of an ongoing epidemiological study into the long-term health effects of CT in young people, [9] aimed to assess whether socio-economic variation exists in young people having these scans in Northern England between 1990 and 2002. If such variation exists, this would imply that certain parts of the population receive higher radiation exposures from medical imaging than others and would provide evidence for the socio-economic variation of medical radiation exposures.

## Methods

Northern England as defined for this study includes the counties of Durham, Cumbria (excluding Barrow-in-Furness in the south), Northumberland, Tyne and Wear and the South Tees area of North Yorkshire. The region includes nine National Health Service (NHS) hospital Trusts, including two neurosurgical centres, which vary in the size of geographical area covered, number and type of patients treated, number and type of hospitals, and the radiology departments' patient loads and services provided. The estimated total population of the region is around 3,000,000, with an estimated 800,000 of these aged under 22 years.

Electronic data, from Radiology Information Systems (RIS), were obtained for patients who had had CT scans in any of the nine Trusts. The retrieved data included patient identifiers, dates of birth and CT examinations, sex, postcode and the type of CT examinations. The data included in this study cover 1990-2002 (the latter being the final date for inclusion of scans in the current ongoing epidemiology study) for patients aged under 22 years at the first CT scan. Complete data were available across the entire study period for two Trusts, including the largest (Newcastle upon Tyne). Six Trusts were missing data for 1991, five for 1992, four Trusts for 1993-1994, three for 1995-1997, two for 1998-1999 and one for 2000. Including an interpolated number of scans for the few hospitals with missing data in the early years of the study period, [9] the estimated missing numbers of patients and scans were both less than 5000. The reasons for or clinical indications from the CT examinations were not recorded on RIS and hence unavailable to this study.

Community-level socio-economic status was defined as the ward-level Townsend deprivation score [15] for each patient. The Townsend score was chosen due to the time period covered by the data included in this study and because, unlike the Index of Multiple Deprivation, it does not include 'health and disability' as a component. Derived from 2001 census data, the Townsend deprivation score is a summary measure consisting of the proportion of households in the area without a car, with more than one person per room and that are not owner-occupied, and also incorporates the number of men (aged 16-64 years) and women (aged 16-59) years who were unemployed at the time of the census. The higher the score, the more deprived the area is assumed. Townsend scores were allocated by linking postcodes to ward-level data. Where postcodes were incomplete or missing, they were obtained using the Royal Mail UK Addresses software (Royal Mail Group Ltd, UK). For addresses where the postcode could still not be ascertained in full or the postcode did not link to a ward code, the mean Townsend score for the smallest relevant postcode unit (sector, district or area) was used commensurate with the information available. Mid-2001 population estimates of numbers of individuals under the age of 21 years in each of the wards included in this study, including those outside the Northern Region, were used to allocate Townsend scores into quintiles (i.e. fifths) according to population size, with quintile 1 representing the wards assumed to be the least deprived and quintile 5 representing those assumed to be the most deprived.

Types of CT examinations were grouped into six categories (head (including 470 scans of the neck), chest, abdomen/pelvis, spine, extremities and miscellaneous or unknown) based on those suggested by Mettler *et al *[16]. The number of scans involving more than one part of the body was small (n = 29) and they were included in the 'miscellaneous' category.

Associations between Townsend deprivation quintiles and other categorical variables were assessed using χ^2 ^tests. Differences in Townsend scores between Trusts were assessed using Kruskal-Wallis tests. Trends in Townsend score in relation to other variables were assessed using Spearman rank correlations. All statistical analyses were done using the statistical software package Stata, version 10 (Stata Corp, College Station, TX, USA).

This study, as part of the larger epidemiological cohort study, was given a favourable ethical opinion by Newcastle and North Tyneside Local Research Ethics Committee and was approved by the National Information Governance Board so as not to require individual-level patient consent.

## Results

A total of 39,676 CT scan records were abstracted from RIS for 21,257 patients aged under 22 years between 1990 and 2002 inclusive. This included 15,957 scans among 8,855 female patients, 23,705 scans among 12,391 male patients, and 14 scans among eleven patients of unknown sex. Direct linkage with Townsend scores was available for 36,609 (92%) scan records, with further Townsend scores added as means from postcode sectors (n = 270), postcode districts (n = 965) and postcode areas (n = 163) to leave 38,007 (96%) scan records eligible for the study. Of the 1669 exclusions, 524 (31%) were addresses in Scotland and 24 (1%) in Northern Ireland with no Townsend scores available for these patients. A further 70 (4%) patients were resident outside the UK. This left a further 956 scan records (2% of all recorded scans) excluded due to missing address information.

Townsend scores significantly varied between the Trusts (p = 0.001), with the highest (i.e. most deprived) scores in patients scanned in South Tyneside and the lowest (i.e. least deprived) in patients scanned in North Cumbria (range of Townsend scores from -4.72 to 18.62). The overall distributions of both scans and patients by quintile of Townsend deprivation scores were significantly different to the distributions of Townsend scores from the census wards included in the study (p < 0.0001) (table 1). Distributions of both scans and patients by Townsend quintiles were similar for males and females, with the proportion of both scans and patients per quintile increasing with increasing area-level deprivation (table 1).

**Table 1:** Numbers of CT scans and individual patients, by quintile of Townsend deprivation and sex, in the Northern Region of England, 1990-2002.

		CT Scans			Patients		
		
Quintile	Townsend range	Total (%)	Males (%)	Females (%)	Total (%)	Male (%)	Females (%)
	
1 (least deprived)	-4.72, -1.43	5782 (15)	3288 (14)	2494 (16)	3091 (16)	1765 (16)	1326 (16)
2	-1.42, 0.70	6702 (18)	4038 (18)	2660 (17)	3353 (17)	1943 (17)	1407 (17)
3	0.70, 3.06	8048 (21)	4792 (21)	3256 (21)	4013 (21)	2302 (20)	1711 (21)
4	3.06, 5.83	8931 (24)	5395 (24)	3532 (23)	4538 (23)	2695 (24)	1840 (23)
5 (most deprived)	5.84, 18.62	8544 (22)	5177 (23)	3364 (22)	4490 (23)	2639 (23)	1848 (23)
Total*	**38007**	**22690**	**15306**	**19485**	**11344**	**8132**	

P for associations between number of scans/patients and quintile of community deprivation all less than < 0.0001.

*11 scans were in 9 patients for whom sex was unknown.

There was a significant association between type of scan and deprivation quintile (p < 0.0001), primarily due to the higher proportions of head scans in the three most deprived quintiles and slightly higher proportions of chest scans and abdomen and pelvis scans in the least deprived groups (table 2).

**Table 2:** Numbers of CT scans by Townsend deprivation quintile and type of examination in patients under 22 years of age in the Northern Region of England, 1990-2002.

	Townsend Quintiles					
Type of examination	1 N (%)	2 N (%)	3 N (%)	4 N (%)	5 N (%)	Total N (%)
Head & neck	4078 (71)	4614 (69)	6010 (75)	6624 (74)	6395 (75)	27721 (73)
Chest	548 (9.5)	691 (10.3)	667 (8.3)	728 (8.2)	705 (8.3)	3339 (8.8)
Abdomen & pelvis	589 (10)	623 (9.3)	646 (8.0)	731 (8.2)	661 (7.7)	3250 (8.6)
Extremities	222 (3.8)	241 (3.6)	222 (2.8)	328 (3.7)	275 (3.2)	1288 (3.4)
Spine	174 (3.0)	280 (4.2)	252 (3.1)	289 (3.2)	306 (3.6)	1301 (3.4)
Miscellaneous/Unknown	89 (1.5)	144 (2.2)	168 (2.1)	130 (1.5)	107 (1.3)	638 (1.7)

**Total**	**5782**	**6702**	**8048**	**8931**	**8544**	**38007**

P for association between quintile of deprivation and type of examination < 0.0001

There was also a significant association (p < 0.0001) between the patient's age at the time of the CT scan and Townsend deprivation quintiles (table 3), with slightly increasing proportions of younger children with increasing deprivation. A similar association with age (p < 0.0001) was seen when restricting the data to include only the first scan of each patient. The number of scans per patient was also associated with Townsend deprivation quintiles (p = 0.014) (table 4). There was a slightly higher proportion of the least deprived patients undergoing only one scan (table 4), although there was no evidence of a trend between deprivation and number of scans (p = 0.37).

**Table 3:** Numbers of CT scans by age and Townsend deprivation quintile in patients under 22 years of age in the Northern Region of England, 1990-2002.

Townsend Quintiles						
Age at time of scan (years)	1 N (%)	2 N (%)	3 N (%)	4 N (%)	5 N (%)	Total N (%)
< 1	420 (7.3)	516 (7.7)	636 (7.9)	812 (9.1)	813 (9.5)	3197 (8.4)
1-4	714 (12)	876 (13)	1045 (13)	1306 (15)	1186 (14)	5127 (13)
5-9	1004 (17)	989 (15)	1209 (15)	1490 (17)	1291 (15)	5983 (16)
10-14	1266 (22)	1509 (23)	1690 (21)	1827 (20)	1830 (21)	8082 (21)
15-19	1940 (34)	2340 (35)	2835 (35)	2880 (32)	2743 (32)	12738 (34)
20-21	438 (7.6)	472 (7.0)	673 (8.4)	616 (6.9)	681 (8.0)	2880 (7.6)

**Total**	**5782**	**6702**	**8048**	**8931**	**8544**	**38007**

P for association between quintile of deprivation and age at time of scan < 0.0001

**Table 4:** Numbers of CT scans per patient, by quintiles of Townsend deprivation scores.

	Townsend Quintiles					
No. of scans	1 N (%)	2 N (%)	3 N (%)	4 N (%)	5 N (%)	Total N (%)
**1**	1924 (62)	1921 (57)	2297 (57)	2640 (58)	2707 (60)	11489 (59)
**2**	730 (24)	857 (26)	1092 (27)	1211 (27)	1133 (25)	5023 (26)
**3**	129 (4.1)	170 (5.1)	184 (4.6)	190 (4.2)	201 (4.5)	874 (4.5)
**4**	133 (4.3)	198 (5.9)	194 (4.8)	221 (4.9)	209 (4.7)	955 (4.9)
**5**	48 (1.6)	38 (1.1)	56 (1.4)	53 (1.2)	57 (1.3)	252 (1.3)
**6**	42 (1.4)	56 (1.7)	63 (1.6)	64 (1.4)	54 (1.2)	279 (1.4)
**7**	15 (0.5)	23 (0.7)	26 (0.7)	30 (0.7)	13 (0.3)	107 (0.6)
**8**	20 (0.7)	25 (0.8)	26 (0.7)	32 (0.7)	40 (0.9)	143 (0.7)
**9**	6 (0.2)	5 (0.2)	10 (0.3)	10 (0.2)	11 (0.2)	42 (0.2)
**10**	11 (0.4)	14 (0.4)	12 (0.3)	21 (0.5)	21 (0.5)	79 (0.4)
**11-20**	21 (0.7)	37 (1.1)	44 (1.1)	53 (1.2)	35 (0.8)	190 (1.0)
**> 20**	12 (0.4)	9 (0.3)	9 (0.2)	13 (0.3)	9 (0.2)	52 (0.3)

**Total**	**3091**	**3353**	**4013**	**4538**	**4490**	**19485**

P for association between quintile of deprivation and number of scans = 0.37

## Discussion

This study suggests that more young patients from the more disadvantaged areas in the Northern England underwent CT scans than would be expected given population distributions of socio-economic status. Further, the proportions of scans were socio-economically varied by age at scan, age at first scan, number of scans and by the body part scanned (with highest numbers of head scans in the more deprived quintiles), all of which influence the level of cumulative excess radiation dose received.

To our knowledge, only one previous study, in Winnipeg, Canada, has investigated associations between socio-economic status and the use of CT scans [17]. An association was seen between socio-economic status and CT usage in adults, but not in children. Socio-economic variation in health is well known across all age groups [1]. Children from disadvantaged areas have been repeatedly reported to suffer injuries requiring presentation at a hospital in the UK [2-6]. This includes a higher rate of 'moderate to severe' head injuries reported in socio-economically deprived children in urban areas, but with the reverse association seen in mixed or rural areas [4].

While injuries do not explain all usage of CT scans, they may contribute to a large proportion of scans in this age group, particularly as the primary association between increased deprivation and increasing use of CT was for scans of the head. Scans of the head have previously been shown to be the most common scan in this age group over a similar time period [9]. In addition, no trend was seen between deprivation and number of scans per patient, which suggests that the socio-economic patterning of radiation exposures due to CT scans is not driven by patients with long-term conditions which may also be related to socio-economic status. A significant association was also seen between deprivation and age at the time of the scan. However, this may reflect a statistical association, rather than one of true importance. Age-group specific analyses were not done when relating number of CT scans to Townsend quintiles because of uncertainties regarding the population sizes and the incomplete geographical coverage over the study period potentially having a noticeable impact on analyses involving relatively small numbers. Further, without data on the reasons for, or clinical indications from, the CT scan, we are unable to assess whether the use of CT, conditional on the presenting injury or disease, differs by socio-economic status.

Since the vast majority of healthcare in the UK, particularly for children, is publicly rather than privately funded, our findings are likely to reflect a true need for diagnosis or treatment, rather than the ability to pay for the scanning procedure. This may be an under-estimate if more advantaged parents are more likely to push for their child to be investigated using a CT scan. However, with increased public understanding of the radiation exposures involved with CT, these parents are the likeliest to ask for an alternative procedure, such as MRI, particularly if their child has previously undergone a CT scan.

Typical radiation doses from CT scans in children and young adults vary with the type of examination [18], but also with a variety of factors including patient size, radiology department and individual practice. The most recent survey of doses from CT in the UK took place in 2003 [19]. A CT scan of the head was estimated to give an effective dose of around 1.5mSv to both children and adults, but an effective dose of 2.5mSv to an infant. Estimated absorbed doses to the brain from such a scan were around 25mGy in infants and children under 6 years, with doses of around 30mGy in older children and adults. Effective doses from chest CT scans are much higher, with estimated effective doses of around 6mSv in both infants and adults and doses of around 4mSv in children. To put this into context, the average annual background radiation dose in the UK is 1.3mSv [20], so, with multiple scans, patients can be exposed to far greater levels of radiation than they would experience under normal circumstances. The use of CT is likely to continue to grow as technology progresses and other clinical applications emerge whereby CT examinations are even more efficient and cost-effective. There needs to be careful consideration of the benefits of using CT for patients, especially for pediatric patients, versus the risks that may be associated with the radiation exposures that these patients receive. CT is highly beneficial in early diagnosis of disease, preventing unnecessary surgery and saving lives. However, to fully establish what the risks to patients are, the results of ongoing empirical epidemiology studies established to directly answer this question are required [21]. In the meantime, CT should only be used where appropriately justified clinically and doses kept as low as reasonably possible.

We obtained RIS data from all nine NHS Trusts in Northern England. The Trusts include a large regional centre with four radiology departments, including one in the largest teaching hospital within the region, a further large teaching hospital and seven smaller Trusts. Although data were incomplete over the entire study period, they were complete for the two largest Trusts in the region and in our previous analysis of temporal trends we estimated that the proportion of missing scans and patients was small [9]. Given the range of Townsend scores seen at all hospitals, it is unlikely that a bias exists due to differing years of data availability between the Trusts. Further, the overall percentages of patients by scan type, age and number of scans included in this study are similar to those for all patients scanned in the Northern England [9], regardless of whether a Townsend score could be allocated. However, as complete population coverage across the study period was not possible, we chose to limit the study to reporting numbers of scans and patients, rather than attempting to calculate rates which would be more uncertain. Although the region has a wide range of socio-economic circumstances, it is, on average, more deprived, in socio-economic terms, than the UK as a whole. Further, a large proportion of the region included in this study is rural, although it includes a small percentage of the population. Previous research in rural areas in Wales has shown that measures of deprivation including car ownership, such as the Townsend deprivation score, are a poor proxy for socio-economic deprivation in sparsely populated rural areas [22]. This is because car ownership is more essential, and therefore less associated with socio-economic deprivation, in these areas than would be expected in more populated urban areas. Different results may have been seen with different measures of socio-economic status, such as the Index of Multiple Deprivation, which includes a 'health and disability' domain, or individual-based measures such as parental occupation, income or education. Therefore, it is possible, though in our view unlikely, that our findings are not representative of socio-economic patterning of CT usage in young people across the entire UK. With ongoing data collection from the rest of the UK, this is an issue that will be addressed in the future.

## Conclusion

Social inequalities exist in the numbers of young people undergoing CT scans with those from deprived areas more likely to do so. This may reflect the rates of injuries in these individuals and implies that certain groups within the population may receive higher radiation doses than others due to medical procedures.

## Competing interests

The authors declare that they have no competing interests.

## Authors' contributions

MSP designed the full epidemiological study with LP and ER, and conceived the idea for this analysis. MSP and JAS analysed the data, KPK provided dosimetry related data. MSP wrote the first draft and all other authors both assisted with interpretation and revised it critically for intellectual content. All authors, except for ER who passed away before the article was finalised, approved the final version. MSP is the guarantor for this article.

## Pre-publication history

The pre-publication history for this paper can be accessed here:

http://www.biomedcentral.com/1472-6963/12/24/prepub
